# Foreign Correspondence

**Published:** 1889-10

**Authors:** 


### Foreign Correspondence.

We are indebted to Dr. Bonwill, who is down on the programme for a demonstration of The Use of Smooth Oval Pointed Pluggers in both the Electrical and Mechanical Mallets for Packing Abbey's Non-Adhesive, as well as any Adhesive or Cohesive Gold Foil, for a report of the meeting of the British Dental Association, taken from the daily press:

"The ninth annual meeting of the British Dental Association opened most pleasantly last evening at Brighton. The president-elect, Mr. S. Lee Rymer, J.P., L.D.S., Eng., held a reception at the Royal Pavilion, and this was followed by a concert and conversazione. There was not a large attendance. The invitations were limited almost entirely to members of the dental and medical professions, and many of those who are expected to put in an appearance during the meeting had not yet arrived in the town. For the meeting is to last to-day, to-morrow, and Saturday, and in these three days some very interesting discussions and demonstrations of some very pleasant social gatherings are to take place. The actual business commences this morning, as early as nine o'clock. Last evening's function was to give the members of the association an opportunity of introduction to the gentleman who is to rule over them during the ensuing year, and of becoming acquainted with one another. The local organizing Honorary Secretary, Mr. J. H. Redman, D.D.S., L.D., S.I., had made fitting arrangements for the reception of so distinguished a body, and the dentists, who journeyed from various parts of the kingdom, were heartily welcomed by their professional brethren of the queen of watering-places. Though, as has been said, the company consisted for the most part of persons associated with dentistry, there were a few others present. Several ladies and several of the medical men of Brighton were there. The formal reception lasted from eight until about a quarter to nine, when the concert commenced in the music-room. The concert was, of course, the chief attraction; not many preferred to roam about the corridor, the north and south drawing-rooms, and the salon, and inspect the many interesting objects,—especially interesting to dentists,—which were exhibited. All the principal makers of dental apparatus and instruments displayed large collections of articles, and many of them were able to show some new invention of most delicate mechanism. Messrs. Coxeter & Son, of Grafton Street, Gower Street, London, for instance, had a small hand-engine for drilling purposes. It is worked by electricity from cells supplied by the Electrical Power Storage Company, and they consider it in many ways preferable to the machine worked with the foot. It certainly seems a very neat contrivance and extremely handy. The same firm also show their most recent form of regulator for controlling the supply of nitrous-oxide gas, and an apparatus for the supply of electric light to assist the dentist in examining the mouth.

The dentist fixes the accumulator upon his forehead and the light is thrown well upon the mouth of the patient. Then Messrs. C. Ash & Sons, of Broad Street, Golden Square, London, and the Dental Manufacturing Company, of Lexington Street, Golden Square, had splendid collections of chairs, engines, apparatus, and every kind of instrument which the dentist could possibly require. The S. S. White Dental Manufacturing Company, Philadelphia, U.S.A., showed a wonderful case of specimens of gold and porcelain crown work by leading American dentists. Messrs. W. & J. Jamieson, of Broad Street, Golden Square, Messrs. G. W. Rutherford & Son, of Poland Street, Oxford Street; Messrs. F. H. Hallam & Son, of Lisson Grove, Marylebone; Messrs. Barth & Co., of Poland Street; Mr. D. Collins, of Poland Street; and Mr. J. F. Blennerhassett, of Vernon Street, King's Cross Road, had also a number of useful instruments on view. A fine collection of micro-photographs illustratiug exostosis and pulp calcification, lent by Mr. D. E. Caush, of Brighton, attracted a great deal of attention. In fact, the whole of the exhibits were closely examined by the professional men, who appeared to be highly gratified with the display. No special decorations had been provided for the occasion. The pavilion rooms were in their ordinary condition, but the visitors were delighted with their appearance and the luxurious accommodation they afforded. It is not in every town that the British Dental Association has a palace in which to hold its annual meeting. A vocal and instrumental concert was given in the dome, the programme being admirably sustained by well-known local talent."

The invited guests, including the ladies of the attending dentists, and also many physicians, numbered many hundred.—Ed.

We are in receipt of a long personal letter from Alfred Burne, D.D.S., Sidney, New South Wales, in which he describes the condition of dentistry in Australia. It seems that it is the common thing for druggists and even manufacturing chemists to run a dental office also. Dr. Burne cites a case where a dispensing apothecary was sued by a patient for alleged ill treatment in relation to a set of teeth which he had inserted for her. His "shop," according to the testimony, was situated back of his drug-store and connected with it. The following circular, considerably abridged, and received from a manufacturing chemist in this city will give some idea of how they do things in Australia. The names of the parties have been changed so as not to give the true parties any free advertising.

We should be most happy to have similar communications from our other *Foreign Correspondents.* This department of the journal can be made one of the most interesting in the journal. Let us hear from all our correspondents, remembering that we must give as well as receive in this world if we desire to keep out of debt. The circular will be found on the next page.—Ed.

